# Vascular hemodynamic effects of penile revascularization surgery and the role of resistive index in follow-up

**DOI:** 10.1186/s12610-024-00243-0

**Published:** 2024-12-20

**Authors:** Fatih Akdemir, Önder Kayigil

**Affiliations:** Faculty of Medicine, Department of Urology, Yıldırım Beyazıt University, Bilkent, Polatlı caddesi, No:125/4, Gazi Mahallesi, Yenimahalle, Ankara, Turkey

**Keywords:** Erectile dysfunction, Penile revascularization, Penile color Doppler Ultrasonography, Resistive index

## Abstract

**Background:**

To evaluate the effects of penile revascularization surgery on penile vascular hemodynamics and to assess the utility of the resistive index (RI) as an objective parameter for postoperative patient follow-up.

**Methods:**

This study included a total of 35 patients who underwent penile revascularization. Penile color Doppler ultrasonography was performed preoperatively and at the third postoperative month to evaluate cavernosal arteries, dorsal arteries, deep dorsal vein, and inferior epigastric artery. During these evaluations, peak systolic velocity, end diastolic velocity, and resistive index were measured. The International Index of Erectile Function questionnaire was administered before surgery and at the third postoperative month. In addition, corpus cavernosum electromyography and cavernosometry tests were performed in all cases preoperatively. Anastomotic patency was assessed using computed tomography angiography at the end of the follow-up period.

**Results:**

The mean preoperative resistive index values were determined to be 0.74 ± 0.07 and 0.73 ± 0.09 cm/s for the right and left cavernosal arteries, respectively, and these values increased to 0.95 ± 0.09 and 0.96 ± 0.06 cm/s, respectively, at the last postoperative control. The mean International Index of Erectile Function-5, 15 scores for the right and left cavernosal arteries were 8.52 ± 4.83 and 19.4 ± 8.54, respectively, preoperatively, and these scores improved to 15.26 ± 4.50 and 35.76 ± 13.65, respectively, at the last postoperative follow-up.

**Conclusion:**

The results of this study suggest that the resistive index can be used as an objective parameter in the diagnosis of erectile dysfunction of vascular origin and in the follow-up and management of the disease following penile revascularization.

**Trial registration:**

NCT06350019/04/03/2024 (retrospectively registered).

**Supplementary Information:**

The online version contains supplementary material available at 10.1186/s12610-024-00243-0.

## Résumé

**Contexte:**

Évaluer les effets de la chirurgie de revascularisation pénienne sur l’hémodynamique vasculaire pénienne et évaluer l’utilité de l’indice résistif (IR) en tant que paramètre objectif pour le suivi postopératoire des patients.

Cette étude a porté sur un total de 35 patients ayant subi une revascularisation pénienne. Une échographie Doppler couleur pénienne a été réalisée en préopératoire et au troisième mois postopératoire pour évaluer les artères caverneuses, les artères dorsales, la veine dorsale profonde et l’artère épigastrique inférieure. Au cours de ces évaluations, la vitesse systolique maximale, la vitesse diastolique finale et l’indice résistif ont été mesurés. Le questionnaire de l’Indice international de la fonction érectile a été administré avant la chirurgie et au troisième mois postopératoire. De plus, des tests d’électromyographie et de cavernosométrie du corps caverneux ont été réalisés en préopératoire dans tous les cas. La perméabilité anastomotique a été évaluée à l’aide d’une angiographie par tomodensitométrie à la fin de la période de suivi.

**Résultats:**

Les valeurs moyennes de l’indice résistif préopératoire ont été déterminées à 0,74 ± 0,07 et 0,73 ± 0,09 cm/s pour respectivement les artères caverneuses droite et gauche, et ces valeurs ont augmenté respectivement à 0,95 ± 0,09 et 0,96 ± 0,06 cm/s lors du dernier contrôle postopératoire. Les scores moyens de l’indice international de la fonction érectile-5, 15 pour les artères caverneuses droite et gauche, étaient respectivement de 8,5 ± 4,8 et 19,4 ± 8,5 avant l’opération ; ces scores se sont améliorés respectivement à 15,2 ± 4,5 et 35,7 ± 13,6 lors du dernier suivi postopératoire.

**Conclusion:**

Les résultats de cette étude suggèrent que l’indice résistif peut être utilisé comme paramètre objectif dans le diagnostic de la dysfonction érectile d’origine vasculaire et dans le suivi et la prise en charge de la maladie suite à une revascularisation pénienne.

**Numéro d’enregistrement de l’essai et date d’inscription:**

NCT06350019/04/03/2024 (enregistré rétrospectivement).

**Mots-clés:**

Dysfonction érectile, Revascularisation pénienne, Échographie Doppler couleur du pénis, Indice résistif

## Introduction

Erectile dysfunction (ED) is defined as the inability to achieve and/or maintain an erection sufficient for satisfactory sexual performance [1]. It has been reported that in more than 70% of cases without endocrine or neurological disorders, ED is of organic origin and is caused by hemodynamic factors such as arterial or venous insufficiency [2]. Penile color Doppler ultrasonography (PCDU), a non-invasive imaging method used to evaluate penile vascular structures, is widely applied in clinical settings. Parameters such as peak systolic velocity (PSV), end-diastolic velocity (EDV), and resistive index (RI) in PCDU hold predictive value for assessing [3]. RI, designed to indicate changes in flow patterns and measure vascular resistance in PCDU, provides clinical insights into vascular resistance and organ perfusion, especially in the examination of small vessels. It is anticipated that RI, in conjunction with erection rigidity value, could help distinguish between arterial and venous insufficiency in the initial evaluation of patients with ED [4]. In this study, patients who underwent penile revascularization were evaluated using PCDU, with measurements of PSV, EDV, and RI, as well as the five- and 15-item International Index of Erectile Function (IIEF-5 and IIEF-15) questionnaires [5] administered both preoperatively and postoperatively. Three months after surgery, the patency of the anastomosis site was confirmed via intravenous contrast-enhanced computed tomography angiography (CTA). This study aimed to evaluate the effects of penile revascularization surgery on penile vascular hemodynamics and to explore the utility of RI as an objective parameter in the postoperative management of ED. In this paper, we present findings from a relatively short follow-up period.

## Materials and methods

Approval for the study was obtained from the local ethics committee (Ankara Yıldırım Beyazıt University approval number: 26379996/91, date: June 18, 2014) All patients were informed about the diagnostic and therapeutic procedures involved in the study, and their informed consent was obtained. A total of 35 patients diagnosed with vascular ED who underwent penile revascularization surgery in our clinic were included. Detailed anamneses of the patients were collected, including age, ED duration, comorbidities that could cause ED, previous trauma, medical or surgical histories, and lifestyle factors. Following a physical examination, the IIEF-5 and IIEF-15 questionnaires were administered. To exclude hypogonadism, levels of follicle-stimulating hormone, luteinizing hormone, total testosterone, and prolactin were measured preoperatively. In addition, PCDU, corpus cavernosum electromyography (CC-EMG), and cavernosometry were performed on all patients in the preoperative period. Patients included were those who had not responded to preoperative treatment with phosphodiesterase 5 inhibitors or intracavernosal alprostadil injections. Patients with a history of urogenital or rectal operations that could affect erectile function, those with penile pathologies such as Peyronie’s disease, and those with neurogenic or psychogenic ED were excluded. At the third postoperative month, the patients were interviewed face-to-face. During these follow-up visits, they were reassessed using the IIEF-5 and IIEF-15 questionnaires. Additionally, PCDU and CTA were performed again.

### PCDU technique

**Fig. 1:**
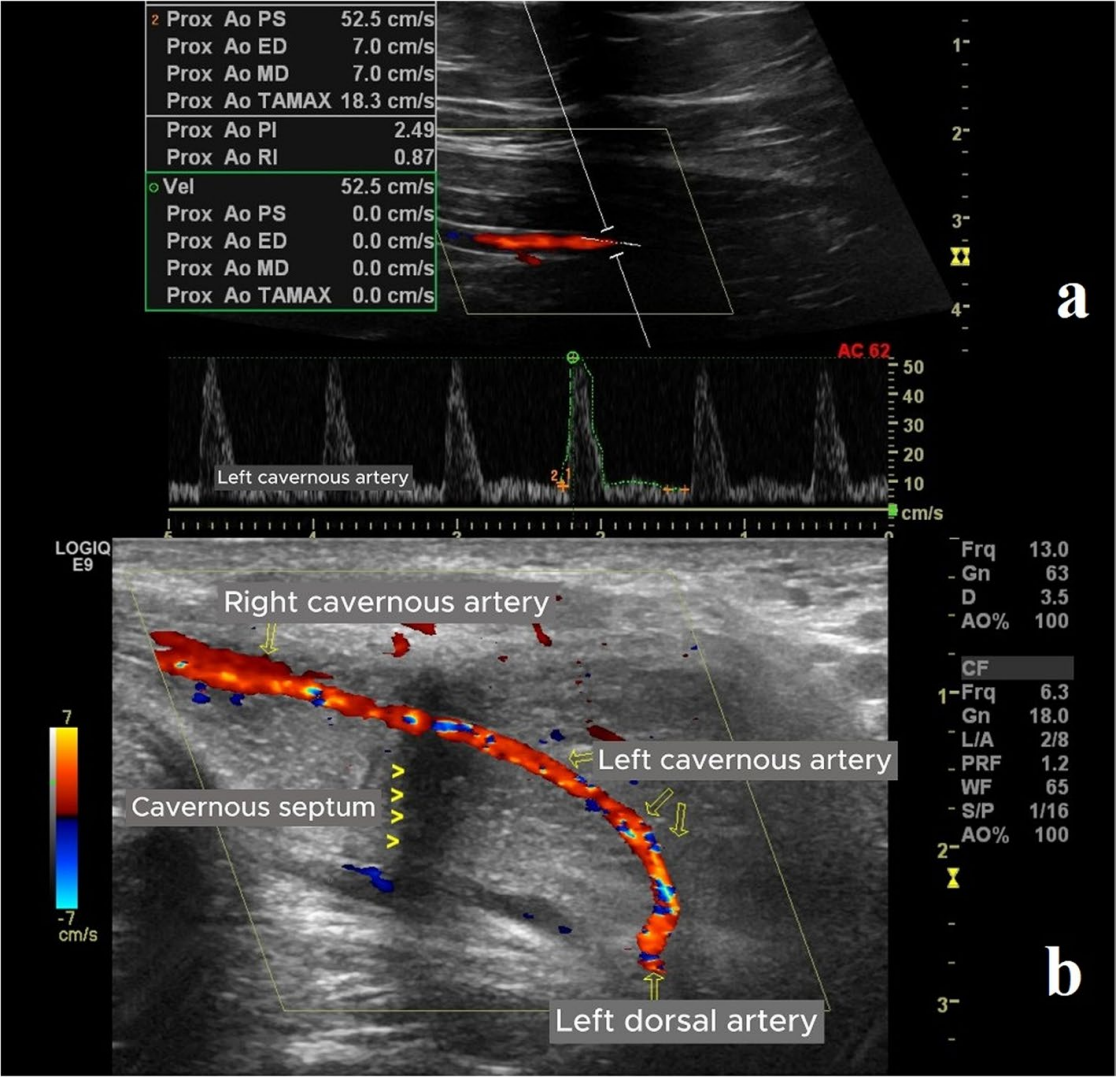
**a**,** b**) PCDU images showing the measurement of penile vascular hemodynamic parameters (PSV, EDV, and RI) after penile revascularization. In the postoperative PCDU, RI increased in correlation with IIEF scores. PS: Peak systolic velocity, ED: End-diastolic velocity, RI: Resistive index, PI: Pulsatility index, MD: Mid-diastolic velocity, PCDU: Penile color Doppler ultrasonography

PCDU was performed in a quiet, comfortable room to optimize patient comfort. To diagnose arterial insufficiency or veno-occlusive disease, PCDU (B-K Medical, Herlev, Denmark) was performed with the patient in a supine position. First, gray-scale imaging of the flaccid penile shaft in both transverse and sagittal planes was used to exclude intracavernosal fibrosis and calcifications. Then, 60 mg of papaverine hydrochloride was injected laterally into one corpus cavernosum using a 22-gauge needle. Twenty minutes after the injection, PCDU was performed using an 8-MHz linear probe angled at approximately 45 degrees. PSV, EDV, and RI values for both cavernosal arteries and the anastomosis region were recorded. Measurements were taken at five-minute intervals for 30 min. Cases with PSV < 25 cm/s were diagnosed with arterial insufficiency, while cases with PSV > 25 cm/s, EDV > 5 cm/s, and RI < 0.85 were considered to have caverno-occlusive disease. RI was calculated using the following formula: RI = (PSV - EDV) / PSV [6]. The patients were advised of the risk of priapism after papaverine hydrochloride injection and instructed to contact the clinic if erection persisted beyond four hours (Fig. 1).

### CC-EMG technique

Penile cavernous electrical activity (CEA) was recorded using a high-speed electromyography module equipped with a computer (Medical Measurement Systems, Enschede, the Netherlands). The sampling frequency was 200 Hz, and a band-pass filter with a cut-off frequency of 0.1–20 Hz was used. For CC-EMG, a monopolar needle electrode was used to measure CEA, with a grounding electrode placed on the patient’s foot to prevent interference from non-penile electrical activity. The electrical activity appeared as a single line on the electromyography recording. CC-EMG recordings were initiated after a 10-minute rest period in a quiet and dimly lit room, and CEA potentials were recorded for 10 min. The CEA potentials of the penile cavernous nerves were assessed by detecting the peak-to-peak amplitudes. Ten minutes later, papaverine hydrochloride (60 mg) was injected into a single cavernous body to avoid discoordination patterns characterized by unchanged or increased CEA following injection, suggesting neurogenic ED. Patients with discoordination patterns were excluded. The relaxation degree was calculated using the following formula: relaxation degree = (pre-injection CEA – post-injection CEA) / pre-injection CEA × 100, as previously described [7]. Patients with relaxation degrees below 50% were excluded from the study.

### Cavernosometry technique

Diagnosis of caverno-occlusive dysfunction was based on the following criteria: (1) a maintenance flow rate exceeding 5 ml/min to sustain an intracavernous pressure of 150 mmHg during the artificial erection test; and (2) a drop in intracavernous pressure of at least 45 mmHg within 30 s after the infusion ended.

### Surgical technique

The operations were performed using a modified version of the Furlow-Fisher technique based on the Virag-V method [8]. Unlike the original Furlow-Fisher procedure, the circumflex collaterals were preserved, and deep dorsal venous valves were not disrupted with a stripper. The inferior epigastric artery was routed to the penile root through a subcutaneous tunnel, and an end-to-side anastomosis with the proximal part of the deep dorsal vein was performed using a 7 − 0 polypropylene suture following the standard microsurgical procedure. The deep dorsal vein was then ligated proximal to the arteriovenous anastomosis to maintain flow. Surgery was performed under optical magnification (x2.5) to prevent neurovascular bundle damage. Postoperatively, the patients were prescribed intravenous heparin (5,000 IU/day) for three days and dipyridamole (75 mg/day) and acetylsalicylic acid (300 mg/day) for three months. They were advised to avoid sexual intercourse for two months.

### CTA technique

At the third postoperative month, the patients underwent CTA. A dose of 60 mg of papaverine hydrochloride was administered to each patient 10 min prior to imaging. A 22-gauge cannula was placed in the basilic or cephalic vein of the patient’s forearm. The patient was placed in a supine position on the imaging stretcher, and the imaging area was identified. Using an automatic injector pump, iodinated contrast material (Iopromide, Ultravist^®^, Schering, Germany) was administered intravenously at a dose of 2 mg/kg and a flow rate of 3 ml/s. Arterial phase pelvic CTA with a 2-mm slice thickness was then performed using a 64-detector, multi-detector computed tomography machine (Aquilion 64, Toshiba^®^, Tokyo, Japan). Following the CTA, sagittal and coronal reformatted images with a 1-mm slice thickness were obtained. The images were evaluated by an experienced radiologist.

## Outcome analysis and statistics

A minimum increase of five points in the IIEF-5 score from the preoperative baseline to the last postoperative visit was considered indicative of improvement (surgical success). Other results were regarded as failure. All statistical analyses were performed using SPSS for Windows (version 21.0, SPSS Inc., Chicago, Illinois, USA). Continuous variables were expressed as mean ± standard deviation values. Student’s t-test was used to compare means, while the chi-square Fisher test was used to compare categorical variables. Categorical data were expressed as numbers and percentages. *P* < 0.05 was considered statistically significant.

## Results

At the time of surgery, the mean age of the patients was 47.17 ± 13.26 (23–69) years, and the mean duration of ED was 32.08 ± 2.13 (29–48) months. Among the patients, 18 (51.4%) were diagnosed with caverno-occlusive disease, 10 (28.5%) with arteriogenic insufficiency, and seven (20.0%) with both caverno-occlusive and arterial pathologies. In addition, nine patients had diabetes mellitus, 16 had a history of smoking, six had hyperlipidemia, and 11 had hypertension. The mean body mass index was determined to be 26.60 ± 2.04 (22–30). The mean total IIEF-15 score was 19.4 ± 8.54 before surgery (*p* < 0.001), which significantly improved to 35.76 ± 13.65 at the third postoperative month. The mean IIEF-5 score increased from 8.52 ± 4.83 preoperatively to 15.26 ± 4.50 at the last follow-up visit (*p* < 0.001). The mean RI values for the right and left cavernosal arteries were 0.74 ± 0.08 and 0.77 ± 0.09, respectively, both of which significantly increased to 0.93 ± 0.07 and 0.94 ± 0.08 (*p* < 0.001) by the third postoperative month. The RI value of the anastomosis area was 0.93 ± 0.09 postoperatively. Table 1 shows the demographic data, preoperative and postoperative vascular parameters, CC-EMG results, and IIEF-5 and IIEF-15 scores. Significant increases were observed in total IIEF-15 and IIEF-5 scores, as well as the RI values of the right and left cavernosal arteries after surgery. In addition, the increase in cavernosal artery RI correlated with IIEF improvements in 71.4% of the patients.

**Table 1:** Demographic parameters and changes in vascular parameters, CC-EMG, IIEF-5 scores, and IIEF-15 scores from the preoperative period to the postoperative period

**Patients (n)**	35		
**Age (year)**	47.17 ± 13.26 (23–69)		
**Comorbidity**			
Diabetes mellitus	9		
Smoking	16		
Obesity (body mass index > 26)	9		
Hypertension or cardiovascular disease	11		
Hyperlipidemia	6		
**Duration of erectile dysfunction **(month)	32.08 ± 2.13 (29–48)		
	**Preoperative**	**Postoperative third month**	**p**
**PCDU **(cm/s)			
**Right cavernosal artery**			
PSV	20.47 ± 3.06	46.10 ± 5.56	<0,001
EDV	5.32 ± 0.65	3.21 ± 0.19	
RI	0.74 ± 0.08	0.93 ± 0.07	
**Left cavernosal artery**			
PSV	24.91 ± 2.42	49.61 ± 7.33	
EDV	5.72 ± 0.59	3.01 ± 0.12	
RI	0.77 ± 0.09	0.94 ± 0.08	
**Anastomosis region**	-		
PSV		48.26 ± 5.95	
EDV		3.31 ± 0.21	
RI		0.93 ± 0.09	
**CC-EMG (**µV)			
Amplitude (before VAD)	271.18 ± 58.34	263 ± 54.16	
Amplitude (after VAD)	81.65 ± 16.41	73.38 ± 12.67	
**IIEF-5**	8.52 ± 4.83	15.26 ± 4.50	
**IIEF-15**	19.4 ± 8.54	35.76 ± 13.65	

*PCDU* Penile color doppler ultrasonography, *PSV* Peak systolic velocity, *EDV* End-diastolic velocity, *RI* Resistive Index, *CC-EMG* Corpus cavernosum electromyography, *VAD* Vasoactive drug, *IIEF* International index of Erectile Function. Statistical analysis: Continuous data are expressed as mean ± standard deviation. Student's t test was used to compare means, and the chi-square Fisher test was used to compare categorical variables

## Discussion

ED is a complex neuropsychological and hormone-mediated vascular process, influenced by both central and peripheral sensory stimuli [9]. Organic ED, which excludes psychogenic causes, can result from vascular, neurogenic, endocrinological, or pharmacological issues [10]. Vascular ED may stem from arterial insufficiency, venous insufficiency, or a combination of both (mixed insufficiency) [11]. Atherosclerosis and endothelial dysfunction are known contributors to ED, with cavernous vessels, due to their small diameter, being affected earlier than coronary vessels. Thus, ED is often an early sign of systemic vascular diseases [12, 13]. In one study, the cardio-ankle vascular index and the ankle-brachial pressure index were measured in individuals with and without ED to assess vascular endothelial damage. The results indicated that the cardio-ankle vascular index was higher in ED cases and inversely correlated with the IIEF score [14]. Another study investigated the relationship between aortic stiffness and ED severity by measuring pulse wave velocity and reported a positive correlation between aortic stiffness and ED severity [15].

According to the Process of Care Consensus Panel, penile vascular surgeries are considered a third-line treatment after the failure of intracavernosal injections [16]. Surgical revascularization techniques include various modifications of anastomosis, such as connecting the inferior epigastric artery to the dorsal artery, to the deep dorsal vein at the penile base, or directly to cavernosal bodies or arteries. Some techniques also employ triple anastomoses, connecting the inferior epigastric artery (end-to-side) with the dorsal penile vein or dorsal penile artery (side-to-side) [17].

PCDU is a non-invasive, dynamic method widely used for evaluating ED. PCDU provides a detailed view of penile vascular anatomy, enabling simultaneous assessment of arterial and venous components. Color imaging enhances Doppler angle adjustment, allowing more accurate measurements of systolic and diastolic velocities [18]. Through PCDU, arterial insufficiency and veno-occlusive dysfunction can be diagnosed. Initial flow rate measurements are typically taken within the first minutes after intracavernosal vasoactive drug injection, but waiting up to 30 min can help reduce anxiety during measurement. Certain indexes based on PSV, EDV, and average velocity have been developed for PCDU to assess flow resistance and pattern changes [19, 20]. These indexes offer advantages by not requiring angular correction or knowledge of vessel dimensions, making them suitable for small or mobile vessels. They provide valuable insights into vascular resistance and organ perfusion [21].

In PCDU, EDV and RI are commonly used to evaluate venous compression. Quam et al. found that an EDV above 5 cm/s indicated venous leakage [22]. Typically, PSV reflects penile cavernosal artery blood flow, EDV reflects venous function [23], and RI indicates vascular resistance and the general condition of the corpus cavernosum, influenced by factors such as peripheral vessel pressure, blood supply to the corpus cavernosum, blood backflow, and overall blood circulation [24]. RI measurement is highly sensitive for distinguishing abnormal waveforms, as its denominator never reaches zero. Xu-Jun Xuan et al. identified a significant positive correlation between RI and penile rigidity in their study, indicating that RI is the most valuable hemodynamic parameter for assessing penile rigidity. Using RI as a quantitatively diagnostic measure for ED could therefore increase diagnostic consistency and accuracy [25].

PCDU studies have established PSV values below 25 cm/s as indicative of arterial insufficiency, while caverno-occlusive dysfunction is typically characterized by a PSV above 25 cm/s, EDV above 5 cm/s, and RI below 0.85 [26, 27]. Although RI has been reported to be a marker of caverno-occlusive dysfunction, Valji and Bookstein determined that RI might also be affected in cases of arterial insufficiency [28]. Factors that constrict the penile arterial system, whether due to organic or neuropsychogenic causes, will increase vascular resistance. Consequently, an RI increase when PSV is below 25 cm/s may indicate either arterial insufficiency or caverno-occlusive dysfunction. In these cases, penile cavernometry remains the gold standard for diagnosing caverno-occlusive dysfunction [29].

Postoperative success in penile revascularization depends on proper anastomotic function, as occlusion from thrombosis or obliteration diminishes surgical efficacy. When PCDU cannot adequately assess anastomotic patency, CTA can be used to confirm patency. In our study, preoperative RI values were found to be 0.74 ± 0.08 for the right and 0.77 ± 0.09 for the left cavernosal artery (both < 0.85). In patients with intact postoperative anastomosis, the mean values RI values for the right and left cavernosal arteries increased to 0.93 ± 0.07 and 0.94 ± 0.08, respectively (both > 0.85). This change in RI suggests improved resistance to flow, i.e., successful prevention of venous leakage and increased cavernous tissue perfusion. We propose that RI, combined with rigidity assessments in the initial evaluation of patients with ED, can assist in distinguishing arterial from venous insufficiency and serve as a useful postoperative evaluation metric. As a result, PCDU may better reveal structural and functional changes in vascular tissue in the corpus cavernosum of patients with ED. Furthermore, the positive correlation between increased RI and IIEF scores in patients with subjective clinical improvement indicates that RI is a cost-effective, non-invasive, and objective method useful for postoperative follow-up evaluations. Although CTA involves contrast material use and radiation exposure, it may be warranted in selected cases where PCDU cannot fully assess anastomotic patency. We also believe that RI may reduce the need for more invasive diagnostic examinations, such as cavernosometry.

### Limitations

This study is limited by the small sample size and relatively short follow-up period. In addition, the techniques applied require high patient compliance and are partially invasive, which may be considered drawbacks.

## Conclusion

Penile revascularization surgery may be recommended for patients with ED who do not respond to oral phosphodiesterase 5 inhibitors and intracavernous injections before considering more invasive options such as penile prosthesis implantation. Our study showed that penile revascularization positively affected penile hemodynamics in vascular ED. RI measured by PCDU is a cost effective, fast, accessible, and non-invasive parameter useful for both diagnosing ED and assessing the postoperative success of penile revascularization surgery. If anastomotic patency cannot be fully evaluated postoperatively via PCDU, CTA can be considered. When selectively applied alongside PCDU and IIEF assessments, CTA makes significant contributions to the management of ED after penile revascularization.

PCDU: Penile color doppler ultrasonography, PSV: Peak systolic velocity, EDV: End-diastolic velocity, RI: Resistive Index, CC-EMG: Corpus cavernosum electromyography, VAD: Vasoactive drug, IIEF: International index of Erectile Function. Statistical analysis: Continuous data are expressed as mean ± standard deviation. Student’s t test was used to compare means, and the chi-square Fisher test was used to compare categorical variables.

## Supplementary Information

Supplementary Material 1.

## Data Availability

No datasets were generated or analysed during the current study.

## Abbreviations

ED: Erectile Dysfunction
PCDU: Penile Color Doppler Ultrasonography
PSV: Peak Systolic Velocity
EDV: End Diastolic Velocity
RI: Resistive Index
IIEF: International Index of Erectile Function
CTA: Computed Tomography Angiography
CC-EMG: Corpus Cavernosum Electromyography
CEA: Cavernous Electrical Activity
