# Supplementary material for: Vascular hemodynamic effects of penile revascularization surgery and the role of resistive index in follow-up
Source: Basic Clin Androl. 2024 Dec 20;34:28. doi: 10.1186/s12610-024-00243-0 (PMC11660643; doi:10.1186/s12610-024-00243-0)
Supplement: Supplementary file 1 — Supplementary Material 1. [file 12610_2024_243_MOESM1_ESM.docx]

TITLE: VASCULAR HEMODYNAMIC EFFECTS OF PENILE REVASCULARIZATION SURGERY AND THE ROLE OF RESISTIVE INDEX IN FOLLOW-UP

SHORT TITLE: VASCULAR EFFECTS OF PENILE REVASCULARIZATION

AUTHORS: FATİH AKDEMİR (MD) (1), ÖNDER KAYIGİL (Prof) (2)

(1): Fatih Akdemir: Yıldırım Beyazıt University Faculty of Medicine, Department of Urology, Bilkent, Ankara, Turkey +90 538 4040494, nfatihakdemir@hotmail.com

ORCID ID: 0009-0006-1735-072X

(2): Önder Kayıgil: Yıldırım Beyazıt University Faculty of Medicine, Department of Urology, Bilkent, Ankara, Turkey +90 5336305764, kayigilo@yahoo.com.tr

ORCID ID: 0000-0001-8261-3940

CORRESPONDING AUTHOR: FATİH AKDEMİR

ADRESS: Polatlı caddesi, No:125/4, Gazi Mahallesi, Yenimahalle, Ankara, Türkiye

TELEPHONE: +905384040494

E-MAİL: nfatihakdemir@hotmail.com

**ABSTRACT**

**Background:** To evaluate the effects of penile revascularization surgery on penile vascular hemodynamics and to assess the utility of the resistive index (RI) as an objective parameter for postoperative patient follow-up.

**Methods:** This study included a total of 35 patients who underwent penile revascularization. Penile color Doppler ultrasonography was performed preoperatively and at the third postoperative month to evaluate cavernosal arteries, dorsal arteries, deep dorsal vein, and inferior epigastric artery. During these evaluations, peak systolic velocity, end diastolic velocity, and resistive index were measured. The International Index of Erectile Function questionnaire was administered before surgery and at the third postoperative month. In addition, corpus cavernosum electromyography and cavernosometry tests were performed in all cases preoperatively. Anastomotic patency was assessed using computed tomography angiography at the end of the follow-up period.

**Results:** The mean preoperative resistive index values were determined to be 0.74±0.07 and 0.73±0.09 cm/s for the right and left cavernosal arteries, respectively, and these values increased to 0.95±0.09 and 0.96±0.06 cm/s, respectively, at the last postoperative control. The mean International Index of Erectile Function-5, 15 scores for the right and left cavernosal arteries were 8.52±4.83 and 19.4±8.54, respectively, preoperatively, and these scores improved to 15.26±4.50 and 35.76±13.65, respectively, at the last postoperative follow-up.

**Conclusion:** The results of this study suggest that the resistive index can be used as an objective parameter in the diagnosis of erectile dysfunction of vascular origin and in the follow-up and management of the disease following penile revascularization.

**Trial registration number and date of registration:** NCT06350019/04/03/2024 (retrospectively registered)

**Keywords:** Erectile dysfunction, Penile revascularization, Penile color Doppler ultrasonography, Resistive index

**INTRODUCTION**

Erectile dysfunction (ED) is defined as the inability to achieve and/or maintain an erection sufficient for satisfactory sexual performance [1]. It has been reported that in more than 70% of cases without endocrine or neurological disorders, ED is of organic origin and is caused by hemodynamic factors such as arterial or venous insufficiency [2]. Penile color Doppler ultrasonography (PCDU), a non-invasive imaging method used to evaluate penile vascular structures, is widely applied in clinical settings. Parameters such as peak systolic velocity (PSV), end-diastolic velocity (EDV), and resistive index (RI) in PCDU hold predictive value for assessing [3]. RI, designed to indicate changes in flow patterns and measure vascular resistance in PCDU, provides clinical insights into vascular resistance and organ perfusion, especially in the examination of small vessels. It is anticipated that RI, in conjunction with erection rigidity value, could help distinguish between arterial and venous insufficiency in the initial evaluation of patients with ED [4]. In this study, patients who underwent penile revascularization were evaluated using PCDU, with measurements of PSV, EDV, and RI, as well as the five- and 15-item International Index of Erectile Function (IIEF-5 and IIEF-15) questionnaires [5] administered both preoperatively and postoperatively. Three months after surgery, the patency of the anastomosis site was confirmed via intravenous contrast-enhanced computed tomography angiography (CTA). This study aimed to evaluate the effects of penile revascularization surgery on penile vascular hemodynamics and to explore the utility of RI as an objective parameter in the postoperative management of ED. In this paper, we present findings from a relatively short follow-up period.

**MATERIAL AND METHODS**

Approval for the study was obtained from the local ethics committee (Ankara Yıldırım Beyazıt University approval number: 26379996/91, date: June 18, 2014) All patients were informed about the diagnostic and therapeutic procedures involved in the study, and their informed consent was obtained. A total of 35 patients diagnosed with vascular ED who underwent penile revascularization surgery in our clinic were included. Detailed anamneses of the patients were collected, including age, ED duration, comorbidities that could cause ED, previous trauma, medical or surgical histories, and lifestyle factors. Following a physical examination, the IIEF-5 and IIEF-15 questionnaires were administered. To exclude hypogonadism, levels of follicle-stimulating hormone, luteinizing hormone, total testosterone, and prolactin were measured preoperatively. In addition, PCDU, corpus cavernosum electromyography (CC-EMG), and cavernosometry were performed on all patients in the preoperative period. Patients included were those who had not responded to preoperative treatment with phosphodiesterase 5 inhibitors or intracavernosal alprostadil injections. Patients with a history of urogenital or rectal operations that could affect erectile function, those with penile pathologies such as Peyronie's disease, and those with neurogenic or psychogenic ED were excluded. At the third postoperative month, the patients were interviewed face-to-face. During these follow-up visits, they were reassessed using the IIEF-5 and IIEF-15 questionnaires. Additionally, PCDU and CTA were performed again.

**PCDU technique:** PCDU was performed in a quiet, comfortable room to optimize patient comfort. To diagnose arterial insufficiency or veno-occlusive disease, PCDU (B-K Medical, Herlev, Denmark) was performed with the patient in a supine position. First, gray-scale imaging of the flaccid penile shaft in both transverse and sagittal planes was used to exclude intracavernosal fibrosis and calcifications. Then, 60 mg of papaverine hydrochloride was injected laterally into one corpus cavernosum using a 22-gauge needle. Twenty minutes after the injection, PCDU was performed using an 8-MHz linear probe angled at approximately 45 degrees. PSV, EDV, and RI values for both cavernosal arteries and the anastomosis region were recorded. Measurements were taken at five-minute intervals for 30 minutes. Cases with PSV < 25 cm/s were diagnosed with arterial insufficiency, while cases with PSV > 25 cm/s, EDV > 5 cm/s, and RI < 0.85 were considered to have caverno-occlusive disease. RI was calculated using the following formula: RI = (PSV - EDV) / PSV [6]. The patients were advised of the risk of priapism after papaverine hydrochloride injection and instructed to contact the clinic if erection persisted beyond four hours (Figure 1).

**CC-EMG technique:** Penile cavernous electrical activity (CEA) was recorded using a high-speed electromyography module equipped with a computer (Medical Measurement Systems, Enschede, the Netherlands). The sampling frequency was 200 Hz, and a band-pass ﬁlter with a cut-off frequency of 0.1–20 Hz was used. For CC-EMG, a monopolar needle electrode was used to measure CEA, with a grounding electrode placed on the patient's foot to prevent interference from non-penile electrical activity. The electrical activity appeared as a single line on the electromyography recording. CC-EMG recordings were initiated after a 10-minute rest period in a quiet and dimly lit room, and CEA potentials were recorded for 10 minutes. The CEA potentials of the penile cavernous nerves were assessed by detecting the peak-to-peak amplitudes. Ten minutes later, papaverine hydrochloride (60 mg) was injected into a single cavernous body to avoid discoordination patterns characterized by unchanged or increased CEA following injection, suggesting neurogenic ED. Patients with discoordination patterns were excluded. The relaxation degree was calculated using the following formula: relaxation degree = (pre-injection CEA – post-injection CEA) / pre-injection CEA × 100, as previously described [7]. Patients with relaxation degrees below 50% were excluded from the study.

**Cavernosometry technique:** Diagnosis of caverno-occlusive dysfunction was based on the following criteria: 1) a maintenance flow rate exceeding 5 ml/min to sustain an intracavernous pressure of 150 mmHg during the artificial erection test; and 2) a drop in intracavernous pressure of at least 45 mmHg within 30 seconds after the infusion ended.

**Surgical technique:** The operations were performed using a modified version of the Furlow-Fisher technique based on the Virag-V method [8]. Unlike the original Furlow-Fisher procedure, the circumﬂex collaterals were preserved, and deep dorsal venous valves were not disrupted with a stripper. The inferior epigastric artery was routed to the penile root through a subcutaneous tunnel, and an end-to-side anastomosis with the proximal part of the deep dorsal vein was performed using a 7-0 polypropylene suture following the standard microsurgical procedure. The deep dorsal vein was then ligated proximal to the arteriovenous anastomosis to maintain flow. Surgery was performed under optical magnification (x2.5) to prevent neurovascular bundle damage. Postoperatively, the patients were prescribed intravenous heparin (5,000 IU/day) for three days and dipyridamole (75 mg/day) and acetylsalicylic acid (300 mg/day) for three months. They were advised to avoid sexual intercourse for two months.

**CTA technique:** At the third postoperative month, the patients underwent CTA. A dose of 60 mg of papaverine hydrochloride was administered to each patient 10 minutes prior to imaging. A 22-gauge cannula was placed in the basilic or cephalic vein of the patient's forearm. The patient was placed in a supine position on the imaging stretcher, and the imaging area was identified. Using an automatic injector pump, iodinated contrast material (Iopromide, Ultravist®, Schering, Germany) was administered intravenously at a dose of 2 mg/kg and a flow rate of 3 ml/s. Arterial phase pelvic CTA with a 2-mm slice thickness was then performed using a 64-detector, multi-detector computed tomography machine (Aquilion 64, Toshiba®, Tokyo, Japan). Following the CTA, sagittal and coronal reformatted images with a 1-mm slice thickness were obtained. The images were evaluated by an experienced radiologist.

**Outcome analysis and statistics**

A minimum increase of five points in the IIEF-5 score from the preoperative baseline to the last postoperative visit was considered indicative of improvement (surgical success). Other results were regarded as failure. All statistical analyses were performed using SPSS for Windows (version 21.0, SPSS Inc., Chicago, Illinois, USA). Continuous variables were expressed as mean ± standard deviation values. Student's t-test was used to compare means, while the chi-square Fisher test was used to compare categorical variables. Categorical data were expressed as numbers and percentages. P < 0.05 was considered statistically significant.

**RESULTS**

At the time of surgery, the mean age of the patients was 47.17 ± 13.26 (23–69) years, and the mean duration of ED was 32.08 ± 2.13 (29–48) months. Among the patients, 18 (51.4%) were diagnosed with caverno-occlusive disease, 10 (28.5%) with arteriogenic insufficiency, and seven (20.0%) with both caverno-occlusive and arterial pathologies. In addition, nine patients had diabetes mellitus, 16 had a history of smoking, six had hyperlipidemia, and 11 had hypertension. The mean body mass index was determined to be 26.60 ± 2.04 (22–30). The mean total IIEF-15 score was 19.4 ± 8.54 before surgery (p < 0.001), which significantly improved to 35.76 ± 13.65 at the third postoperative month. The mean IIEF-5 score increased from 8.52 ± 4.83 preoperatively to 15.26 ± 4.50 at the last follow-up visit (p < 0.001). The mean RI values for the right and left cavernosal arteries were 0.74 ± 0.08 and 0.77 ± 0.09, respectively, both of which significantly increased to 0.93 ± 0.07 and 0.94 ± 0.08 (p < 0.001) by the third postoperative month. The RI value of the anastomosis area was 0.93 ± 0.09 postoperatively. Table 1 shows the demographic data, preoperative and postoperative vascular parameters, CC-EMG results, and IIEF-5 and IIEF-15 scores. Significant increases were observed in total IIEF-15 and IIEF-5 scores, as well as the RI values of the right and left cavernosal arteries after surgery. In addition, the increase in cavernosal artery RI correlated with IIEF improvements in 71.4% of the patients.

**DISCUSSION**

ED is a complex neuropsychological and hormone-mediated vascular process, influenced by both central and peripheral sensory stimuli [9]. Organic ED, which excludes psychogenic causes, can result from vascular, neurogenic, endocrinological, or pharmacological issues [10]. Vascular ED may stem from arterial insufficiency, venous insufficiency, or a combination of both (mixed insufficiency) [11]. Atherosclerosis and endothelial dysfunction are known contributors to ED, with cavernous vessels, due to their small diameter, being affected earlier than coronary vessels. Thus, ED is often an early sign of systemic vascular diseases [12,13]. In one study, the cardio-ankle vascular index and the ankle-brachial pressure index were measured in individuals with and without ED to assess vascular endothelial damage. The results indicated that the cardio-ankle vascular index was higher in ED cases and inversely correlated with the IIEF score [14]. Another study investigated the relationship between aortic stiffness and ED severity by measuring pulse wave velocity and reported a positive correlation between aortic stiffness and ED severity [15].

According to the Process of Care Consensus Panel, penile vascular surgeries are considered a third-line treatment after the failure of intracavernosal injections [16]. Surgical revascularization techniques include various modifications of anastomosis, such as connecting the inferior epigastric artery to the dorsal artery, to the deep dorsal vein at the penile base, or directly to cavernosal bodies or arteries. Some techniques also employ triple anastomoses, connecting the inferior epigastric artery (end-to-side) with the dorsal penile vein or dorsal penile artery (side-to-side) [17].

PCDU is a non-invasive, dynamic method widely used for evaluating ED. PCDU provides a detailed view of penile vascular anatomy, enabling simultaneous assessment of arterial and venous components. Color imaging enhances Doppler angle adjustment, allowing more accurate measurements of systolic and diastolic velocities [18]. Through PCDU, arterial insufficiency and veno-occlusive dysfunction can be diagnosed. Initial flow rate measurements are typically taken within the first minutes after intracavernosal vasoactive drug injection, but waiting up to 30 minutes can help reduce anxiety during measurement. Certain indexes based on PSV, EDV, and average velocity have been developed for PCDU to assess flow resistance and pattern changes [19, 20]. These indexes offer advantages by not requiring angular correction or knowledge of vessel dimensions, making them suitable for small or mobile vessels. They provide valuable insights into vascular resistance and organ perfusion [21].

In PCDU, EDV and RI are commonly used to evaluate venous compression. Quam et al. found that an EDV above 5 cm/s indicated venous leakage [22]. Typically, PSV reflects penile cavernosal artery blood flow, EDV reflects venous function [23], and RI indicates vascular resistance and the general condition of the corpus cavernosum, influenced by factors such as peripheral vessel pressure, blood supply to the corpus cavernosum, blood backflow, and overall blood circulation [24]. RI measurement is highly sensitive for distinguishing abnormal waveforms, as its denominator never reaches zero. Xu-Jun Xuan et al. identified a significant positive correlation between RI and penile rigidity in their study, indicating that RI is the most valuable hemodynamic parameter for assessing penile rigidity. Using RI as a quantitatively diagnostic measure for ED could therefore increase diagnostic consistency and accuracy [25].

PCDU studies have established PSV values below 25 cm/s as indicative of arterial insufficiency, while caverno-occlusive dysfunction is typically characterized by a PSV above 25 cm/s, EDV above 5 cm/s, and RI below 0.85 [26, 27]. Although RI has been reported to be a marker of caverno-occlusive dysfunction, Valji and Bookstein determined that RI might also be affected in cases of arterial insufficiency [28]. Factors that constrict the penile arterial system, whether due to organic or neuropsychogenic causes, will increase vascular resistance. Consequently, an RI increase when PSV is below 25 cm/s may indicate either arterial insufficiency or caverno-occlusive dysfunction. In these cases, penile cavernometry remains the gold standard for diagnosing caverno-occlusive dysfunction [29].

Postoperative success in penile revascularization depends on proper anastomotic function, as occlusion from thrombosis or obliteration diminishes surgical efficacy. When PCDU cannot adequately assess anastomotic patency, CTA can be used to confirm patency. In our study, preoperative RI values were found to be 0.74 ± 0.08 for the right and 0.77 ± 0.09 for the left cavernosal artery (both < 0.85). In patients with intact postoperative anastomosis, the mean values RI values for the right and left cavernosal arteries increased to 0.93 ± 0.07 and 0.94 ± 0.08, respectively (both > 0.85). This change in RI suggests improved resistance to flow, i.e., successful prevention of venous leakage and increased cavernous tissue perfusion. We propose that RI, combined with rigidity assessments in the initial evaluation of patients with ED, can assist in distinguishing arterial from venous insufficiency and serve as a useful postoperative evaluation metric. As a result, PCDU may better reveal structural and functional changes in vascular tissue in the corpus cavernosum of patients with ED. Furthermore, the positive correlation between increased RI and IIEF scores in patients with subjective clinical improvement indicates that RI is a cost-effective, non-invasive, and objective method useful for postoperative follow-up evaluations. Although CTA involves contrast material use and radiation exposure, it may be warranted in selected cases where PCDU cannot fully assess anastomotic patency. We also believe that RI may reduce the need for more invasive diagnostic examinations, such as cavernosometry.

**Limitations:** This study is limited by the small sample size and relatively short follow-up period. In addition, the techniques applied require high patient compliance and are partially invasive, which may be considered drawbacks.

**CONCLUSION**

Penile revascularization surgery may be recommended for patients with ED who do not respond to oral phosphodiesterase 5 inhibitors and intracavernous injections before considering more invasive options such as penile prosthesis implantation. Our study showed that penile revascularization positively affected penile hemodynamics in vascular ED. RI measured by PCDU is a cost effective, fast, accessible, and non-invasive parameter useful for both diagnosing ED and assessing the postoperative success of penile revascularization surgery. If anastomotic patency cannot be fully evaluated postoperatively via PCDU, CTA can be considered. When selectively applied alongside PCDU and IIEF assessments, CTA makes significant contributions to the management of ED after penile revascularization.

**List of abbreviations**

**ED:** Erectile Dysfunction

**PCDU:** Penile Color Doppler Ultrasonography

**PSV:** Peak Systolic Velocity

**EDV:** End Diastolic Velocity

**RI:** Resistive Index

**IIEF:** International Index of Erectile Function

**CTA:** Computed Tomography Angiography

**CC-EMG:** Corpus Cavernosum Electromyography

**CEA:** Cavernous Electrical Activity

**Statement and Declarations**

**Funding:** The authors declare that no funds, grants, or other support were received during the preparation of this manuscript.

**Competing interests:** The authors have no relevant financial or non-financial interests to disclose.

**Author contributions:** Both authors contributed to the study conception and design. Material preparation, data collection, and analysis were performed by FA and ÖK. The first draft of the manuscript was written by FA and ÖK, and both authors commented on previous versions. Critical revisions of the manuscript were performed by FA and ÖK. Both authors have read and approved the final manuscript.

**Ethics approval:** This study was conducted in accordance with the principles of the Declaration of Helsinki. Approval was granted by the Ethics Committee of Ankara Yıldırım Beyazıt University Faculty of Medicine.

**Approval number:** 26379996/91, date: June 18, 2014).

**ClinicalTrials.gov Identifier:** NCT06350019

**Consent to participate:** Informed consent was obtained from all individual participants included in the study.

**Consent to publish:** The authors affirm that informed consent for publication of the images in the figures was obtained from all human research participants.

**Acknowledgements:** None

**REFERENCES**

1. Shafik A, Shafik I, El Sıbai O, Shafik AA. On the pathogenesis of penile venous leakage: role ofthe tunica albuginea. BMC Urol 2007; 5;7:14.  doi: 10.1186/1471-2490-7-14.
2. Zang Z, Deng C. The use of color Doppler in the diagnosis of erectile dysfunction: Zhonghua Nan Ke Xue 2004; 10:298-301. PMID: 15148931
3. Aversa A, Sarteschi LM. The role of penile colour-dupplex ultrasound for the evaluation of erectile dysfunction. J Sex Med 2007; 4:1437-47.  doi: 10.1111/j.1743-6109.2007.00546.x.
4. Chiou RK, Pomeray BD, Chen WS, Anderson JC, Wobig RK and Taylor RJ. Hemodynamic patterns of pharmacologically induced erection: Evaluation by color doppler sonography. J.Urol 1998; 159:109-16. doi: 10.1016/s0022-5347(01)64028-x.
5. Rosen RC, Riley A, Wagner G, [Osterloh IH](https://www.ncbi.nlm.nih.gov/pubmed/?term=Osterloh%20IH%5BAuthor%5D&cauthor=true&cauthor_uid=9187685), [Kirkpatrick J](https://www.ncbi.nlm.nih.gov/pubmed/?term=Kirkpatrick%20J%5BAuthor%5D&cauthor=true&cauthor_uid=9187685), [Mishra A](https://www.ncbi.nlm.nih.gov/pubmed/?term=Mishra%20A%5BAuthor%5D&cauthor=true&cauthor_uid=9187685). The International Index of erectile function (IIEF). A multidimensional scale for assessment of erectile function. Urology 1997; 49:822-30. doi: 10.1016/s0090-4295(97)00238-0.
6. [Benson](https://pubmed.ncbi.nlm.nih.gov/?term=Benson+CB&cauthor_id=8416651) CB, [Aruny](https://pubmed.ncbi.nlm.nih.gov/?term=Aruny+JE&cauthor_id=8416651) JE, [Vickers Jr](https://pubmed.ncbi.nlm.nih.gov/?term=Vickers+MA+Jr&cauthor_id=8416651) MA. Correlation of duplex sonography with arteriography in patients with erectile dysfunction. AJR Am J Roentgenol 1993; 160:71-3. doi: 10.2214/ajr.160.1.8416651.
7. Kayıgil O, Metin A. Relaxation degree: a new concept in erectile dysfunction. Int Urol Nephrol 2001; 33:391-4. doi: 10.1023/a:1015230216682.
8. Furlow WL, Fisher J, Knoll LD. Current status of penile revascularization with deep dorsal vein arterialization: experience with 95 patients. Int J Impot Res 1990; 2:348.
9. Rogers RS, Graziottin TM, Lin CS, Kan YW, Lue TF. Intracavernosal Vascular Endothelial Growth Factor (VEGF) injection and adeno-associated virus-mediated VEGF gene therapy prevent and reverse venogenic erectile dysfunction in rats. Int J Impot Res 2003; 15:26-37. doi: 10.1038/sj.ijir.3900943.
10. Gollijanin D, Singer E, Davis R, Bhatt S, Seftel A, Dogra V. Doppler evaluation of erectile dysfunction – part 1. Int J Impot Res 2007; 19:37-42. doi: 10.1038/sj.ijir.3901477.
11. Yıldırım D, Bozkurt IH, Gürses B, Çırakoğlu A. A new parameter in the diagnosis of vascular erectile dysfunction with penile doppler ultrasound: cavernous artery ondulation index. Eur Rew Med Pharmacol Sci 2013; 17:1382-8. PMID: 23740453
12. Gandaglia G, Briganti A, Jackson G, Kloner RA, Montorsi F, Montorsi P et al. A systematic review of the association between erectile dysfunction and cardiovascular disease. European Urology 2014; 65:968-78. [doi.org/10.1016/j](https://doi.org/10.1016/j). eururo.2013.08.023
13. Wan ZH, Zhang YJ, Chen L, Guo YL, Li GH, Wu D et al. G protein-coupled receptor kinase 2 inhibition improves erectile function through amelioration of endothelial dysfunction and oxidative stress in a rat model of type 2 diabetes. Asian Journal of Andrology 2019; 21:74–9. [doi.org/10.4103/aja.aja_69_18](https://doi.org/10.4103/aja.aja_69_18)
14. Bulbul E, Aydin E, Yilmaz E. Evaluation of endothelial dysfunction with cardio-anklevascular index measurements in patients with erectiledysfunction. Andrology 2022; 10:926-30. DOI: 10.1111/andr.13191
15. Demirelli E, Karagöz A, Öğreden E, Oğuz U, Vural A, Aksu M et al. The relationship between the severity of erectile dysfunction and aortic stiffness. Andrologia 2020;00:e13544. DOI: 10.1111/and.13544
16. The process of care model for evaluation and treatment of erectile dysfunction. Process of Care Consensus Panel. Int J Impot Res 1999; 11:59. doi:10.1038/sj.ijir.3900411.
17. Virag F, Zwang G, Dermange H, Legman M. Vasculogenic impotence: A review of 92 cases with 54 surgical operations. Vas Surg 1981; 15:9-17.
18. Mancini M, Bartolini M, Maggi M, Innocenti P. and Forti G. The presence of arterial anatomical variations can affect the results of duplex sonographic evaluation of penile vessels in impotent patients. J Urol 1996; 155:1919-25.
19. Nelson R, Pretorius DH. The Doppler signal: Where dose it come from and what does it mean? AJR 1988; 151:439-47. doi:10.2214/ajr.151.3.439.
20. Peter N, Burns PhD. The Pysical principles of Doppler and spectral analysis. J Clin Ultrasound 1987; 15:567-90. doi:10.1002/jcu.1870150903.
21. Shamloul R: Peak systolic velocities may be falsely low in young patients with erectile dysfunction. J Sex Med 2006; 3:138-43. doi:10.1111/j.1743-6109.2005.00136.x.
22. Quam JP, King BF, James EM, Lewis RW, Brakke DM, Ilstrup DM, et al. Duplex and color sonographic evaluation of vasculogenic impotence. A J Roentgenol. 1989; 153:1141-8. doi:10. 2214 /ajr . 153.6.1141.
23. Demir O, Demir T, Kefi A, Secil M, Comlekci A, [Yesil](https://pubmed.ncbi.nlm.nih.gov/?term=Yesil+S&cauthor_id=19322005) S et al. Penile vascular impairment in erectile dysfunction patients with metabolic syndrome: penile Doppler ultrasound findings. Urol Int 2009; 82:175-8.  doi: 10.1159/000200795
24. Kuo YC, Liu SP, Chen JH, Chang HC, Tsai VF, [Ju-Ton Hsieh](https://pubmed.ncbi.nlm.nih.gov/?term=Hsieh+JT&cauthor_id=19912490). Feasability of a novel audio-video sexual stimulation system: an adjunct to the use of penile duplex Doppler ultrasonography for the investigation of erectile dysfunction. J Sex Med 2010; 7:3979-83. doi: 10.1111/j.1743-6109.2009.01583.x.
25. [Xuan](https://www.ncbi.nlm.nih.gov/pubmed/?term=Xuan%20XJ%5BAuthor%5D&cauthor=true&cauthor_uid=25994651) XJ, [Bai](https://www.ncbi.nlm.nih.gov/pubmed/?term=Bai%20G%5BAuthor%5D&cauthor=true&cauthor_uid=25994651) G, [Zhang](https://www.ncbi.nlm.nih.gov/pubmed/?term=Zhang%20CX%5BAuthor%5D&cauthor=true&cauthor_uid=25994651) CX, [Xu](https://www.ncbi.nlm.nih.gov/pubmed/?term=Xu%20C%5BAuthor%5D&cauthor=true&cauthor_uid=25994651) C, [Lu](https://www.ncbi.nlm.nih.gov/pubmed/?term=Lu%20FD%5BAuthor%5D&cauthor=true&cauthor_uid=25994651) FD, [Peng](https://www.ncbi.nlm.nih.gov/pubmed/?term=Peng%20Y%5BAuthor%5D&cauthor=true&cauthor_uid=25994651) Y et al. The application of color doppler flow imaging in the diagnosis and therapeutic effect evaluation of erectile dysfunction. [Asian J Androl](https://www.ncbi.nlm.nih.gov/pmc/articles/PMC4736339/) 2016; 18:118-22. doi: 10.4103/1008-682X.155533.
26. Mihmanli I, Kantarci F. Erectile dysfunction. Semin Ultrasound CT MR. 2007; 28:274-86. doi: 10.1053/j. sult. 2007.05.004
27. Golijanin D, Singer E, Davis R, Bhatt S, Seftel A, Dogra V. Doppler evaluation of erectile dysfunction-part 2. Int J Impot Res 2007; 19:43-48. doi: 10.1038/sj.ijir.3901478.
28. Valji K, Bookstein JJ. Diagnosis of Arteriogenic Impotence: Efficacy of Duplex Sonography as a Screening Tool. American Roentgen Ray Society 1993; 16:65-9. doi: 10.2214/ajr.160.1.8416650.
29. Bella AJ, Brant WO, Lue TF. Penile anatomy. In: Bertolotto M, editör. Color Doppler US of the Penis. Berlin: Springer; Pp. 2012; 11-4.

**Figure legends**

**Figure 1: a, b)** PCDU images showing the measurement of penile vascular hemodynamic parameters (PSV, EDV, and RI) after penile revascularization. In the postoperative PCDU, RI increased in correlation with IIEF scores. PS: Peak systolic velocity, ED: End-diastolic velocity, RI: Resistive index, PI: Pulsatility index, MD: Mid-diastolic velocity, PCDU: Penile color Doppler ultrasonography

**Table legends**

**Table 1:** Demographic parameters and changes in vascular parameters, CC-EMG, IIEF-5 scores, and IIEF-15 scores from the preoperative period to the postoperative period.

| **Patients (n)** | 35 | | |
| --- | --- | --- | --- |
| **Age (year)** | 47.17 ± 13.26 (23–69) | | |
| **Comorbidity**  Diabetes mellitus  Smoking  Obesity (body mass index > 26)  Hypertension or cardiovascular disease  Hyperlipidemia | 9  16  9  11  6 | | |
| **Duration of erectile dysfunction** (month) | 32.08 ± 2.13 (29–48) | | |
|  | **Preoperative** | **Postoperative third month** | **p** |
| **PCDU** (cm/s)  **Right cavernosal artery**  PSV  EDV  RI  **Left cavernosal artery**  PSV  EDV  RI  **Anastomosis region**  PSV  EDV  RI | 20.47 ± 3.06  5.32 ± 0.65  0.74 ± 0.08  24.91 ± 2.42  5.72 ± 0.59  0.77 ± 0.09  - | 46.10 ± 5.56  3.21 ± 0.19  0.93 ± 0.07  49.61 ± 7.33  3.01 ± 0.12  0.94 ± 0.08  48.26 ± 5.95  3.31 ± 0.21  0.93 ± 0.09 | <0,001 |
| **CC-EMG (**µV)  Amplitude (before VAD)  Amplitude (after VAD) | 271.18 ± 58.34  81.65 ± 16.41 | 263 ± 54.16  73.38 ± 12.67 |  |
| **IIEF-5** | 8.52 ± 4.83 | 15.26 ± 4.50 |  |
| **IIEF-15** | 19.4 ± 8.54 | 35.76 ± 13.65 |  |

PCDU: Penile color doppler ultrasonography, PSV: Peak systolic velocity, EDV: End-diastolic velocity, RI: Resistive Index, CC-EMG: Corpus cavernosum electromyography, VAD: Vasoactive drug, IIEF: International index of Erectile Function. Statistical analysis: Continuous data are expressed as mean ± standard deviation. Student's t test was used to compare means, and the chi-square Fisher test was used to compare categorical variables.
